# Associations of body mass index and gestational weight gain with term pregnancy outcomes in urban Cameroon: a retrospective cohort study in a tertiary hospital

**DOI:** 10.1186/s13104-015-1765-9

**Published:** 2015-12-19

**Authors:** Florent Ymele Fouelifack, Jeanne Hortence Fouedjio, Jovanny Tsuala Fouogue, Zacharie Sando, Loic Dongmo Fouelifa, Robinson Enow Mbu

**Affiliations:** Obstetrics and Gynecology Unit of Yaoundé Central Hospital, Department of Obstetrics and Gynecology, Higher Institute of Medical Technology of Nkolondom, Yaoundé, Cameroon; Research, Education and Health Development Group “GARES-Falaise” Dschang-Cameroon, P.O. Box: 31186, Yaoundé, Cameroon; Department of Obstetrics and Gynecology of the Faculty of Medicine and Biomedical Sciences of University of Yaoundé 1, Obstetrics and Gynecology Unit of the Yaoundé Central Hospital, Yaoundé, Cameroon; Obstetrics and Gynecology unit of the Douala Gynaeco-Obstetric and Pediatric Hospital, Douala, Cameroon; Department of Anatomy and Morphological Sciences of the Faculty of Medicine and Biomedical Sciences of the University of Yaoundé 1, P.O. Box 1364, Yaoundé, Cameroon; Faculty of Medicine, School of Armies Health Services of Lomé, University of Lomé, P.O. Box: 14148, Lomé, Togo

**Keywords:** Pregnancy, Body mass index, Outcome, Complications, Obesity, Gestational weight gain, IOM recommendations, Cameroon

## Abstract

**Background:**

Obesity is a rising public health issue worldwide. Guidelines regarding maternal body mass index (BMI) and gestational weight gain (GWG) are missing in Cameroon where maternal mortality rate remains very high. We hypothesized that obesity and inappropriate GWG are associated with poor pregnancy outcomes. We aimed at assessing associations of BMI and GWG with pregnancy outcomes.

**Methods:**

This was a retrospective cohort study at the Yaoundé Central Hospital. We included women with term singleton deliveries in the post-partum ward. The World Health Organisation classification of BMI and the United States Institute Of Medicine (IOM) categories of GWG were used to stratify participants. Poor maternal outcome was defined by the occurence of caesarean section, preeclampsia or obstetrical haemorrhage. Poor perinatal outcome was defined by the occurence of perinatal death, admission in intensive care unit, low birth weight, macrosomia or fifth minute Apgar score <7. Multiple logistic regressions were used to calculate unadjusted and adjusted Odds Ratios (uOR, aOR) for poor maternal outcome (PMO) and for poor perinatal outcome (PPO) in each category of BMI and GWG. Adjustment was done for age, scarred uterus, sickle cell disease, malaria, human immunodeficiency virus (HIV) infection, parity and smoking.

**Results:**

Of the 462 participants, 17 (4 %) were underweight (BMI < 18.5), 228 (49 %) had normal pre-pregnancy BMI, 152 (33 %) were overweight (25 ≤ BMI < 30) and 65 (14 %) were obese (BMI ≥ 30). Following the IOM recommendations, GWG was normal for 186 (40 %) participants, less than recommended for 131 (28 %) and above the recommended norms for 145 (32 %). GWG above the IOM recommendation was significantly associated with PMO (aOR: 1.7, 95 % CI 1.1–2.8). GWG less than the IOM recommended values, overweight and obesity were not significantly associated with poor pregnancy outcomes.

**Conclusion:**

While waiting for local recommendations for GWG, the IOM recommendations can be used for Cameroonian women as far as maternal outcome is concerned. Unlike in studies in different ethnic and racial groups, abnormal BMI was not associated with poor pregnancy outcomes in our cohort of Cameroonian women.

## Background

Obesity is a rising threat to health worldwide [1]. The trends are the same in Cameroon with marked differences between rural and urban populations on one hand and between men and women on the other hand, the latter being more affected [2–4]. In 2002, the prevalence of obesity among Cameroonian urban women was estimated at 17 % [3, 4]. It is therefore obvious that the proportion of women entering pregnancy with overweight or obesity will increase. This will constitute a challenge to the health system.

Association between pre-pregnancy obesity and maternal or neonatal morbidity has been demonstrated in developed countries as early as 1945 [5, 6]. Several pregnancy complications have been shown to be significantly associated with pre-pregnancy overweight and obesity: miscarriage [7], gestational diabetes [8], induction of labor [8], cesarean section [8, 9], post partum hemorrhage [8], preeclampsia [8, 10], dysfunctional labor (slower labor progress from 4 to 10 cm) [11], stillbirths [12, 13], neonatal death [13], macrosomia [14] and admission into neonatal intensive care unit [8]. To our knowledge only one study evaluating pregnancy outcomes in Cameroonian obese women has been published [15]. That study found that 64 % of obese women had severe complication [uterine atony (56 %), placenta retention (50 %), placenta accreta (2 %), and cesarean Sect. (21 %)]. The same study reported that 63 % of neonates from obese women had severe complications [macrosomia (11 %), still birth (4 %), poor Apgar score (26 %), intra-partum or early neonatal deaths (13 %)].

Weight gain during pregnancy is attributable to the uterus and its contents (foetus, amniotic fluid and placenta), breasts, blood and interstitial fluid. A smaller fraction of that weight gain is due to an increase in cellular water and deposition of new fat and protein (maternal reserves) [16, 17]. Though the range of weight gain considered normal is wide, it depends on the pre-gestational body mass index (BMI) [17]. The most widely accepted recommendations for gestational weight gain (GWG) are those issued by the Institute of Medicine (IOM) of the United States of America (Table 1) [18]. There are special populations for which the IOM recommendations are not fully applicable (adolescents, women with multiple foetuses, minority racial and ethnic groups). The rationale behind those guidelines was that pregnancy outcomes are better in women gaining weight within recommended range during pregnancy than in other women. Full implementation of the Institute of Medicine GWG recommendations may reduce obstetric risk, reduce post-partum weight retention, normalize infant birth weight, and improve long-term health [18]. Before undertaking such a public health measure, a pre-requisite is to assess the validity of the IOM in Cameroonian women. To our knowledge, only two studies have compared outcomes of pregnancies between Cameroonian women who had GWG within the IOM recommended range and those who did not [19, 20]. The following complications were significantly higher in women with excessive GWG (as per IOM) than in those with recommended GWG: pre-eclampsia, induction of labor, prolonged labor, episiotomy, instrumental delivery, cesarean section, post-partum hemorrhage, acute fetal distress, mal-presentation, macrosomia, birth trauma and poor Apgar score at the first minute (110 overweight women compared to 110 controls) [19]. The second study (231 participants) revealed that the prevalence of macrosomia was significantly higher in case of excessive GWG [20]. Given the scarcity of data on the effects of pre-pregnancy BMI and GWG on pregnancy outcomes in Cameroonian women we carried out this study to fill that knowledge gap. Our objectives were to compare pregnancy outcomes in women with abnormal pre-pregnancy weight to that in women with normal weight (following WHO classification of BMI) and to compare pregnancy outcomes in women with abnormal gestational weight gain to that in women with normal gestational weight gain (following recommendations by the IOM).

**Table 1:** The American Institute of Medicine Gestational Weight Gain Recommendations [18]

Pre-pregnancy weight categories	Ranges of body mass index (kg/m^2^)	Recommended total weight (kg)
Underweight	<18.5	12.5–18
Normal weight	18.5–24.9	11.5–16
Overweight	25–29.9	7–11.5
Obese	≥30	5–9

This table shows the 2009 recommendations of gestational weight gain in each class of body mass index

*kg* kilogram, *m* ^*2*^ square meter

## Methods

We carried out a retrospective cohort study at the Yaoundé Central Hospital (YCH). This is a tertiary and teaching hospital in the capital of Cameroon. We included all women after a singleton term delivery from January 2, 2014 to April 30, 2014. We excluded women with incomplete files, unknown pre-pregnancy weight, severe physical conditions and those who did not consent. Sampling was consecutive.

### Measurements of exposure and outcome variables

We first explained the purpose and the procedure of the study to all women in the post-partum ward. We then invited women to sign the consent form. A confidential pretested technical form was used to collect data. Socio-demographic and baseline data were collected on the questionnaire [age, marital status, height, occupation, pre-pregnancy weight (self reported)] and from obstetric records (parity, gestational age at delivery, and weight at the end of pregnancy). Maternal complications were retrieved from obstetric records: *hyperemesis gravidarum*, malaria, preeclampsia, ante-partum hemorrhage, post-partum hemorrhage, cesarean section, augmentation of labour, gestational age at delivery and non cephalic presentation at birth. Foetal and neonatal complications were retrieved from obstetric records: macrosomia, low birth weight, early neonatal death, intra-uterine death, intra-partum death, acute foetal distress, admission in intensive care unit, Apgar scores at the first and fifth minute. Potential confounders were retrieved from obstetric records: human immunodeficiency virus (HIV) infection, uterine scar, smoking status, malaria in pregnancy and sickle cell disease.

### Statistical analysis

The sample size was calculated with the software Stata^®^ (version 13). The probability on type 1 error was 5 % and the power was 99 %. The minimal sample size was 151, but we included all eligible women during the study period. Microsoft Excel^®^ (version 2010) was used to compile data. We measured association between continuous variables (BMI and GWG) and a binary variable (pregnancy outcome). Adjustment for potential confounders (HIV infection, uterine scar, smoking status, malaria in pregnancy and sickle cell disease) was also done and a multivariable logistic regression was applied. In the first model, independent variable was the pre-pregnancy BMI. In the second model GWG was the independent variable. For both models, we had two categorical outcome variables: poor maternal outcome (PMO) and poor perinatal outcome (PPO). PMO was defined by the presence of at least one of the following complications: cesarean section, preeclampsia and obstetrical haemorrhage. The presence of at least one of the following defined PPO: perinatal death, admission into the intensive care unit, low birth weight (birth weight <2500 grams), macrosomia (birth weight ≥4000 grams) and fifth minute Apgar score <7. In the first model, PMO and PPO in subgroups with abnormal BMI were compared to the subgroup with normal BMI. In the second model, PMO and PPO in subgroups with abnormal GWG were compared to those in the subgroup with recommended GWG. Unadjusted and adjusted odds ratios (uOR and aOR) were computed.

### Ethical considerations

Clearance was obtained from the ethical committee of University of Yaoundé 1. Authorization was obtained from YCH staff. Written informed consent was compulsory. Confidentiality was observed. For participants <16 year old written informed consent was obtained from a guardian.

## Results

We had 462 participants. The mean age of participants was 27.6 ± 5.9 with extremes of 13 and 43 years.

### Clinical characteristics and distribution of participants by BMI

The distribution of participants by clinical characteristics in each class of BMI is described in Table 2.

**Table 2:** Distribution of participants according to clinical characteristics and BMI

Clinical characteristics	Prevalence		Maternal BMI (kg/m^2^)			
	No	%	<18.5	18.5–24.9	25.0–29.9	≥30.0
Total	462	100	17	228	152	65
Age (years)						
<20	38	8	4	25	8	1
20–34	366	79	12	183	122	49
≥35	58	13	1	20	22	15
Smoking						
Yes	31	7	1	14	9	7
No	431	93	16	214	143	58
Parity						
0	184	40	8	104	55	17
≥1	278	60	9	124	97	48
HIV infection						
Yes	31	67	0	18	11	2
No	431	93	17	210	141	63
Uterine scar						
Yes	53	11	0	24	13	16
No	409	89	17	204	139	49
Sickle cell disease						
Yes	11	2	0	6	5	0
No	451	98	17	222	147	65

*No* absolute number of observations, *HIV* human immunodeficiency virus

Seventy-nine per cent of participants were between 20 and 34 year old. HIV prevalence was 7 %. Multiparous women accounted for 60 % and women with normal BMI accounted for 49.35 % of participants.

Out of the 462 participants, 17 (4 %) were underweight (BMI < 18.5 kg/m^2^), 228 (49 %) had normal BMI (18.5 ≤ BMI ≤ 24.9), 152 (33 %) were overweight (25 ≤ BMI ≤ 29.9) and 65 (14 %) were obese (BMI ≥ 30). Most of the participants (79 %) were between 20 and 34 years old. Of the 462 participants, 31 (7 %) were smokers, 184 (40 %) were primiparous and 31 (7 %) were HIV infected. Sickle cell disease was found in 11 (3 %) participants and 53 (11 %) had a scarred uterus.

GWG was within IOM 2009 recommendations for 186 (40 %) participants. GWG was below the IOM 2009 recommendations in 131 (28 %) and above in 145 (32 %) participants.

### Clinical characteristics and distribution of participants by GWG

Table 3 shows the distribution of participants according to clinical characteristics in each category of the IOM 2009 recommendations for GWG. Forty per cent of participants had normal GWG as per IOM. There was no significant difference in the prevalence of clinical characteristics among the different classes of GWG.

**Table 3:** Distribution of participants according to clinical socio-characteristics and GWG

Clinical characteristics	Prevalence		GWG (kg)		
	No	%	GWG < IOM rec.	GWG = IOM rec.	GWG > IOM rec.
Total	462	100	131	186	145
Age (years)					
<20	38	8	18	15	5
20–34	366	79	103	147	116
≥35	58	13	10	24	24
Smoking					
Yes	31	7	2	23	6
No	141	93	129	163	139
Parity					
0	184	40	66	63	55
≥1	278	60	65	123	90
HIV infection					
Yes	31	7	12	13	6
No	431	93	119	173	139
Uterine scar					
Yes	53	11	12	25	16
No	409	899	119	161	129
Sickle cell disease					
Yes	11	2	2	5	4
No	451	98	129	181	141

*No* absolute number of observations, *HIV* human immunodeficiency virus

### Prevalence of maternal and perinatal complications in different classes of BMI

Table 4 shows distribution of maternal and perinatal complications in different classes of BMI.

**Table 4:** Prevalence of maternal and perinatal complications according to BMI

Complications	Prevalence		BMI (kg/m^2^)			
	No	%	<18.5	18.5–24.9	25.0–29.9	≥30.0
Total	462	100	17 (%)	228 (%)	152 (%)	65 (%)
Maternal complications						
Cesarean section	176	38	4 (24)	81 (36)	63 (41)	28 (43)
Obstetrical hemorrhage	51	11	1 (6)	24 (11)	20 (13)	6 (9)
Dynamic dystocia	78	17	2 (12)	44 (19)	18 (12)	14 (22)
Pre-eclampsia	51	11	1 (6)	24 (11)	20 (13)	6 (9)
Poor maternal outcome	206	45	4 (24)	97 (43)	73 (48)	32 (49)
Perinatal complications						
Admission ICU	62	13	4 (24)	33 (14)	16 (11)	9 (14)
5th min Apgar score <7	44	910	2 (12)	21 (9)	14 (9)	7 (11)
Low birth weight	51	11	5 (30)	23 (10)	17 (11)	6 (9)
Macrosomia	33	7	0	15 (7)	12 (8)	6 (9)
Perinatal death	23	5	0	8 (4)	9 (6)	6 (9)
Poor neonatal outcome	131	28	6 (35)	62 (27)	44 (29)	19 (29)

*kg* kilograms, *GWG* gestational weight gain, *IOM* Institute of Medicine, *rec.* recommendations, *ICU* intensive care unit

The most frequent maternal complication was cesarean section which was found in 38 % of participants. Pre-eclampsia and obstetrical hemorrhage affected 11 % of participants each. The prevalence of admission in pediatric intensive care unit (ICU) was 13 % while 11 % of our newborns had low birth weight.

### Prevalence of maternal and perinatal complications in different categories of GWG

Rates of maternal and perinatal complications in each class of GWG among participants are detailed in Table 5.

**Table 5:** Prevalence of specific maternal and perinatal complications and outcomes according to Gestational Weight Gain (GWG)

Complications	Prevalence		GWG (kg)		
	No	%	GWG < IOM rec.	GWG = IOM rec.	GWG > IOM rec.
Total	462	100	131 (%)	186 (%)	145 (%)
Maternal complications					
Cesarean section	176	38	34 (26)	69 (37)	73 (50)
Obstetrical hemorrhage	51	11	14 (11)	19 (10)	18 (12)
Dynamic dystocia	78	17	31(24)	30 (16)	17 (12)
Pre-eclampsia	51	11	14 (11)	19 (10)	18 (12)
Poor maternal outcome	206	45	44 (34)	80 (43)	82 (57)
Perinatal complications					
Admission ICU	62	13	20 (15)	26 (14)	16 (11)
5th min Apgar score <7	44	10	14 (11)	19 (10)	11 (8)
Low birth weight	51	11	16 (12)	25 (13)	10 (7)
Macrosomia	33	7	6 (5)	11 (6)	16 (11)
Perinatal death	23	5	7 (5)	5 (3)	11 (8)
Poor perinatal outcome	131	28	36 (27)	54 (29)	41 (28)

*kg* kilograms, *GWG* gestational weight gain, *IOM* Institute of Medicine, *rec.* recommendations, *ICU* intensive care unit

The prevalence of PMO was 45 % and that of PPO was 28 %. No significant difference was found between rates of complications among different classes of GWG.

### Measures of associations between GWG, BMI and pregnancy outcomes

Unadjusted and adjusted odds ratios expressing associations between GWG and PMO and PPO are shown in Table 6. It also shows associations between BMI and PMO and PPO. BMI was neither associated with PMO nor with PPO. Excessive GWG was associated with PMO (aOR: 1.7; 95 % CI 1.1–2.8).

**Table 6:** Associations of BMI and GWG with PMO and PPO

Exposures and confounders	PMO	PPO
	aOR (95 % CI)	aOR (95 % CI)
Body mass index		
Underweight (BMI < 18.5)	0.5 (0.2–1.6)	1.5 (0.5–4.3)
Overweight (25 ≤ BMI ≤ 29.9)	0.8 (0.5–1.5)	1.1 (0.6–1.9)
Obesity (BMI ≥ 30)	0.7 (0.4–1.3)	0.9 (0.5–1.9)
Normal weight (18.5 ≤ BMI ≤ 25)	1.0	1.0
Gestational weight gain		
<Recommended GWG	0.6 (0.4–1.1)	0.9 (0.6–1.8)
>Recommended GWG	*1.7* (*1.1–2.8*)	0.9 (0.6–1.6)
Recommended GWG	1.0	1.0
Age	1.3 (0.78–2.0)	1.2 (0.7–1.9)
Smoking	0.5 (0.8–2.0)	1.1 (0.5–2.6)
Malaria	1 (0.7–1.5)	0.9 (0.5–1.3)
Sickle cell disease	3.7 (0.9–4.8)	1.0 (0.3–3.9)
Uterine scar	*4.9* (*2.4–9.7*)	1.7 (0.9–3.3)
Parity	0.9 (0.6–1.3)	0.9 (0.6–1.5)
HIV infection	*0.3* (*0.1–0.8*)	0.6 (0.2–1.5)

*aOR* adjusted odds ratio, *Vs* versus, *GWG* gestational weight gain, *BMI* body mass index, *PPO* poor perinatal outcome, *PMO* poor maternal outcome

Women with GWG above IOM recommendations were at a higher risk (1.7 fold) of having PMO (caesarean section, obstetrical haemorrhage or preeclampsia) are highlighted in italic

## Discussion

Our study found that 4 % (17 out of 462) of women were underweight while 14 % (65 out of 462) were obese. Overweight women accounted for 33 % (152 out of 462) of the study population. Pre-pregnancy BMI was normal for 49 % (228 out of 462) of our participants. This distribution is in accordance with a study among pregnant women by Mbu et al. and with an anthropometric survey among urban adults by Kamadjeu et al. in Cameroon [2, 19]. Indeed, they found that 50 % of urban women were either obese or overweight. The proportion of underweight women in our sample (4 %) was inferior to that reported (7 %) by Kamadjeu et al. [2]. An explanation is that the prevalence of underweight reported in their study was highest above 55 year old (out of reproductive ages) while 79 % (366 out of 462) of our participants were aged 20–34 year old. Moreover the latest demographic and health survey conducted in Cameroon (2011) revealed that 7 % (1064 out of 15 426) of women of reproductive age are underweight, the prevalence being highest among those aged 15–19 year old [21]. The same survey reported that the national prevalence of excessive BMI (obesity and overweight) among women of reproductive age in Cameroon was 32 %. Figures were higher for educated (42 % among the most educated) and wealthy women (47 % among the richest stratum) who are more likely to live in urban areas [21]. We had similar findings.

GWG was below the IOM recommendations for 131 (28 %) and above for 145 (31 %) of our participants. These figures appear excessive and raise the question of suitability of the IOM recommendations for our population. Our sample was not representative of the whole population of the country. Participants were recruited in a referral hospital known to have a higher proportion of maternal and foetal complications than the general population. This selection bias added to the fact that even in the USA, almost 1 in 3 women had weight gain outside the IOM recommendations makes that restraint unlikely [17]. The IOM recommendations have been criticized for too narrow limits [22]. A study on sample of 1 849 Asian women found that 38 % of pre-pregnancy underweight and 31 % of pre-pregnancy normal BMI women gained less than IOM recommendation [23]. In that study 52 % of overweight women and 64 % of obese women gained more than the recommended weight [23]. A large scale study of 56 101 pregnant women in Europe revealed that 74 % of overweight nulliparous women had GWG above the IOM recommendations [24]. Another study in America concluded that out of 5377 women 69 % had GWG out of IOM recommendations [24].

The prevalence of complications was quite high in our sample. The rate of cesarean section was 38 % (176 out of 462). This rate was 26 % among women with GWG below the IOM recommendations, 37 % in those with recommended GWG and 50 % in women with excessive GWG. Similar findings have been reported by Crane et al. [25]. The setting in which the study was conducted is a major referral hospital in the town. This can explain very high rates of cesarean deliveries. Nevertheless, rates of cesarean delivery tend to increase with excessive GWG and to reduce with GWG below the IOM recommendations. Rates of obstetrical hemorrhage (either ante-partum or post-partum hemorrhage) and pre-eclampsia were almost the same in all classes of GWG. This could be explained by the high proportions of primiparous women in each class of GWG (50 % in excessive GWG, 34 % in recommended GWG and 38 % in GWG below IOM recommendations) in our sample. Indeed it has been proven that primiparous women have a 3.1 relative risk of developing pre-eclampsia [26]. Using that composite, we found that GWG below the IOM recommendations was protective against PMO (uOR: 0.5; 95 % CI 0.4–0.80) but not significantly after adjustment (aOR: 0.6; 95 % CI 0.4–1.1) the effect size being small. Excessive GWG was significantly associated with PMO (aOR: 1.7; 95 % CI 1.1–2.8). In previous studies on smaller samples in Cameroon, without adjustment, excessive GWG was significantly associated with pre-eclampsia, cesarean delivery, prolonged labor and postpartum hemorrhage with big effect sizes [19, 20]. We did adjustment for the following confounders which, taken alone had big effect sizes on maternal outcome: smoking, sickle cell disease, uterine scar, and HIV infection (Table 6). We also adjusted for age, malaria during pregnancy and parity though they had little effect sizes. In a systematic review, the strength of associations of GWG with pre -eclampsia and cesarean delivery has been shown to be dependent upon age, parity, race and ethnicity; Researchers were therefore advised to properly address confounders [27].

No significant differences were found between classes of GWG for macrosomia, admission into pediatric intensive care unit (ICU), poor (<7) 5th minute Apgar score, low birth weight and perinatal death. Total rate of admission in paediatric ICU (13 %) and perinatal deaths (5 %) found in this study were consistent with the high infant mortality rate that prevails in the country [21]. We observed a low birth weight rate of 11 % which is in line with the national average of 10 % [21]. Though the rate of PPO was high (28 %) in our study, no association was found with abnormal GWG (Table 6). Some studies found significant association between GWG and adverse perinatal outcomes (macrosomia, low birth weight, large/small-for-gestational age, intra-partum fetal death) but other did not [19, 24, 25, 28]. A systematic review concluded that strong evidence supported association between GWG and macrosomia, low birth weight and large/small-for-gestational age [27].

We found that pre-pregnancy BMI was not significantly associated with pregnancy outcomes (Table 6). This is different from what has been reported by several researchers who worked on larger samples [6, 8, 9, 10, 11, 12, 13, 14, 29]. Nonetheless the proportion of PMO was 43 % among women with normal BMI, 48 % among overweight women and 49 % among obese women. This could have been significant in a sample with more participants. Future researches should better explore this issue with larger samples of Cameroonian women.

The main strength of our study is to stand as the first (to our knowledge) to assess pregnancy outcome in all categories of BMI and GWG in Cameroon. Limitations include the following selection bias: our sample was from a cosmopolitan urban area but did not represent all Cameroonian pregnant women. To fully assess the IOM recommendations, a sample representative of all ethnic groups, age, parity, environment and social strata found in the country must be studied. We used hospital-based data of a tertiary center. This hospital is a referral center and therefore has a higher rate of materno-foetal complications than primary health care settings. Rates of pregnancy complications in a population-based study might be more close to their prevalence in the general population. Retrospective collection of data (especially weight) may lead to a certain degree of inaccuracy.

## Conclusion

One-third of women had GWG above the IOM recommendations and there was no difference of proportions following pre-pregnancy BMI. GWG above IOM recommendations was significantly associated with poor maternal outcome but not with poor foetal outcome. No significant association was found between pre-pregnancy BMI and poor materno-foetal outcomes. Prospective population-based cohort studies are needed to further explore association between BMI and GWG with pregnancy outcomes before recommendations are formulated for Cameroonian women.

## Abbreviations

aOR: Adjusted odds ratios
BMI: Body mass index
GWG: Gestational weight gain
HIV: Human immunodeficiency virus
ICU: Intensive care unit
IOM: Institute of Medicine
PMO: Poor maternal outcome
PPO: Poor perinatal outcome
u OR: Unadjusted odds ratios
USA: United States of America
YCH: Yaoundé Central Hospital
